# 5-Benzyl­idene-3-phenyl-2-phenyl­imino-1,3-thia­zolidin-4-one

**DOI:** 10.1107/S1600536811023658

**Published:** 2011-06-25

**Authors:** Matthias Zeller, Vijay Satam, Ravi Kumar Bandi, Ajaya Kumar Behera, Bijay Kumar Mishra, Hari Pati, Moses Lee

**Affiliations:** a Department of Chemistry, Youngstown State University, Youngstown, Ohio 44555, USA; b Division of Natural and Applied Sciences, and Department of Chemistry, Hope College, Holland, MI 49423, USA; c Department of Chemistry, Sambalpur University, Jyoti Vihar 768 019, Sambalpur, Orissa, India

## Abstract

The title compound, C_22_H_16_N_2_OS, is a chalcone analog with a thia­zolidinone core that was synthesized as a potential cytotoxic and anti­cancer agent. The structure is commensurately modulated by unit-cell doubling along the direction of the *a* axis of the cell. The two crystallographically independent mol­ecules are differerentiated by the dihedral angle between the mean planes of the benzyl­idene phenyl group against the thia­zolidin-4-one moiety, which is 5.01 (7)° in one mol­ecule, and 17.41 (6)° in the other. The two mol­ecules are otherwise close to being indistinguishable and are related by crystallographic pseudo-translation. The two mol­ecules are not planar but are slightly bent with the benzyl­idene and phenyl­imino substituents being bent upwards with respect to the center planes of the two mol­ecules. The degree of bending of the two halves of the thia­zolidin-4-one moieties (defined as the planes that inter­sect at the S atom) are 11.08 (7) and 15.88 (7)°. Packing of the mol­ecules is facilitated by C—H⋯π inter­actions and slipped π–π stacking between one of the phenyl rings and a neighboring ethylene π system [distance between the centroid of the ethylene group and the closest phenyl C atom = 3.267 (2) Å, *Cg*(phenyl)⋯*Cg*(ethylene) = 3.926 Å].

## Related literature

Abdel-Aziz *et al.* (2010 ▶), Babu *et al.* (2011 ▶) and Chavda *et al.* (2009 ▶) describe the use of conjugated styryl ketones and related compounds as potential cytotoxic and anti­cancer agents. Satam *et al.* (2011 ▶) gives background to compounds with a thia­zolidinone pharmacophore and describe structures related to the title compound.
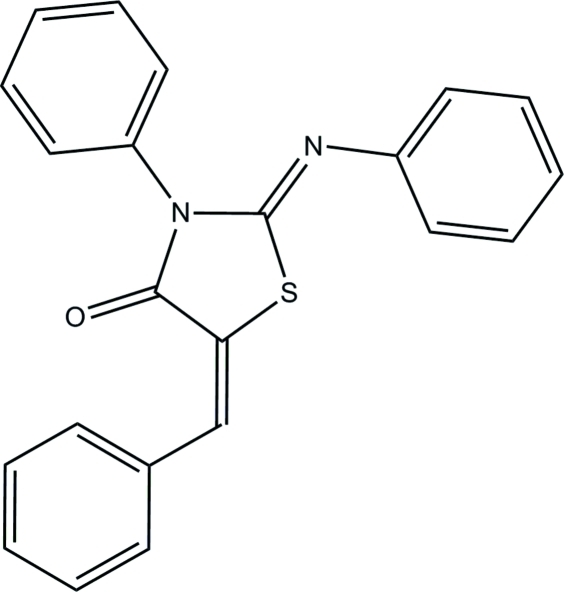


## Experimental

### 

#### Crystal data

C_22_H_16_N_2_OS
*M* *_r_* = 356.43
Monoclinic, 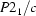

*a* = 10.7814 (9) Å
*b* = 32.779 (3) Å
*c* = 9.8907 (8) Å
β = 98.392 (1)°
*V* = 3458.0 (5) Å^3^
*Z* = 8
Mo *K*α radiation
μ = 0.20 mm^−1^
*T* = 100 K
0.55 × 0.41 × 0.33 mm

#### Data collection

Bruker SMART APEX CCD diffractometer
Absorption correction: multi-scan (*SADABS*; Bruker, 2009 ▶) *T*_min_ = 0.632, *T*_max_ = 0.746
23712 measured reflections
10081 independent reflections
7732 reflections with *I* > 2σ(*I*)
*R*_int_ = 0.025

#### Refinement

*R*[*F* ^2^ > 2σ(*F* ^2^)] = 0.045
*wR*(*F* ^2^) = 0.125
*S* = 1.03
10081 reflections
469 parameters
H-atom parameters constrained
Δρ_max_ = 0.46 e Å^−3^
Δρ_min_ = −0.23 e Å^−3^

### 

Data collection: *APEX2* (Bruker, 2009 ▶); cell refinement: *SAINT* (Bruker, 2009 ▶); data reduction: *SAINT*; program(s) used to solve structure: *SHELXTL* (Sheldrick, 2008 ▶); program(s) used to refine structure: *SHELXTL*; molecular graphics: *SHELXTL* and *Mercury* (Macrae *et al.*, 2008 ▶); software used to prepare material for publication: *SHELXTL* and *publCIF* (Westrip, 2010 ▶).

## Supplementary Material

Crystal structure: contains datablock(s) I, global. DOI: 10.1107/S1600536811023658/gk2385sup1.cif

Structure factors: contains datablock(s) I. DOI: 10.1107/S1600536811023658/gk2385Isup2.hkl

Supplementary material file. DOI: 10.1107/S1600536811023658/gk2385Isup3.cml

Additional supplementary materials:  crystallographic information; 3D view; checkCIF report

## Figures and Tables

**Table 1:** Hydrogen-bond geometry (Å, °)

*D*—H⋯*A*	*D*—H	H⋯*A*	*D*⋯*A*	*D*—H⋯*A*
C12*A*—H12*A*⋯S1*B*	0.95	2.92	3.6214 (15)	131
C12*A*—H12*A*⋯C2*B*	0.95	2.85	3.7340 (19)	156
C12*A*—H12*A*⋯C3*B*	0.95	2.59	3.5270 (19)	167
C12*B*—H12*B*⋯S1*A*^i^	0.95	2.96	3.6118 (15)	127
C12*B*—H12*B*⋯C3*A*^i^	0.95	2.76	3.6700 (19)	162

Symmetry code: (i) 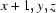.

**Fig. 1.**
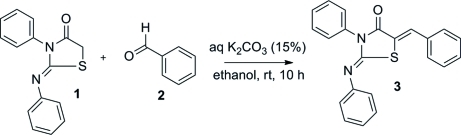
Synthetic pathway towards 5-benzylidene-3-phenyl-2-(phenylimino)thiazolidin-4-one (3)

**Fig. 2.**
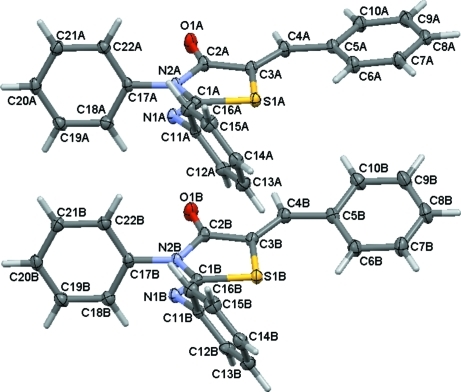
Thermal ellipsoid style plot of the molecules of the title compound with atom numbering scheme. Probability levels for non-H atoms are at 50%.

**Fig. 3.**
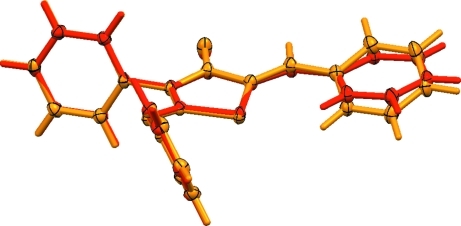
Least squares overlay of the two crystallographically independent molecules. Red: molecule A, orange: molecule B.

**Fig. 4.**
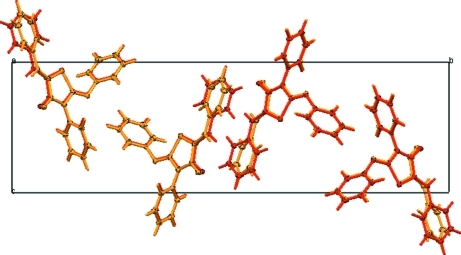
View down the a-axis showing the pseudotranslation along this axis. Red: molecule A, orange: molecule B.

**Fig. 5.**
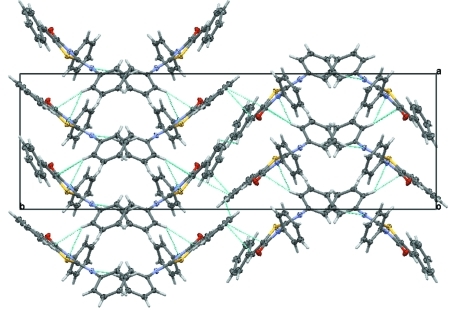
Packing view of (3). Blue dashed lines indicate significant C—H···π interactions.

## supplementary crystallographic information

### Comment

Continuing our work on the design, syntheses, and evaluation of conjugated styryl ketones and related compounds as potential cytotoxic and anticancer agents (Chavda *et al.*, 2009, Babu *et al.*, 2011, Satam *et al.*, 2011) we synthesized a series of novel chalcone analogs possessing a thiazolidinone core. We envisaged that a combination of the 3-aryl-2-propenoyl unit in chalcones and the thiazolidinone pharmacophore would lead to a series of novel chalcone analogs that may provide a synergistic effect to exhibit interesting cytotoxic activities against malignant cells. A novel series of eleven compounds was assessed for cytotoxic activity against murine B16 and L1210 cancer cell lines (Satam *et al.*, 2011). The title compound belonging to this series was subjected to X-ray crystallographic analysis in order to investigate the geometry about the alkene bond as well as the overall conformation of the compound.

The title compound (3) was synthesized by condensation of 2-phenylimino-3-phenylthiazolidin-4-one (1) with benzaldehyde as shown in Figure 1. Re-crystallization from methanol yielded crystals suitable for X-ray diffraction analysis. The structure crystallizes in *P*2_1_/c with two crystallographically independent but chemically identical molecules A and B per asymmetric part of the unit cell, Figure 2. Bond distances and angles in both moleucles are in the expected ranges, a *Mogul* geometry check as implemented in the program Mercury (Macrae *et al.*, 2008) did not indicate any unusual geometric parameters, and double and single bonds are located as expected (Scheme 1, Figure 1). The benzylidene double bond shows the phenyl substituent and the sulfur atoms to be in *cis* position to each other. The two molecules have very similar conformations and in both molecules the heterocylic ring sections are not planar but are slightly U-shaped with the benzylidene and phenylimino substituents being bent upwards with respect to the center planes of the two molecules. The degree of bending of the thiazolidin-4-one moieties, defined as the angle between the plane formed by the two N atoms, the S atom and C1 and C17 on the one hand and that of the carbonyl group, the sulfur atom and C3 and C4 on the other, is 11.08 (7)° for the A molecule, and 15.88 (7)° for the B molecule. A similar slight deviation from planarity was observed earlier for the 4-methyl benzylidene derivative of the title compound, which has an equivalent bend angle of 15.9 (1)°. The 3-bromo-6 methyl derivative, on the other hand, is essentially planar with a bend angle of only 2.98 (4)° (Satam *et al.*, 2011).

The major difference between the two molecules in the structure of the title compound is the rotation angle of the benzylidene phenyl rings with respect to the remainder of the molecules. The torsion angle of the C—H bonded phenyl group against the thiazolidin-4-one moiety is 5.01 (7)° in molecule A, and 17.41 (6)° in molecule B. All other torsion angles, that of the phenylimino and of the phenyl rings, differ only marginally between the two molecules as can be seen in an overlay of the two molecules, Figure 3.

The two crystallographically independent molecules are not only conformationally very similar, they are also related by a crystallographic pseudotranslation along the *a*-axis of the unit cell as shown in Figure 4. The structure can indeed also be successfully refined in a smaller cell with the *a*-axis length cut in half. If refined in this smaller volume setting the benzylidene phenyl ring has to be refined as being disordered over two equally occupied mutually incompatible sites closely resembling the overlays shown in Figures 3 and 4. *R* values and figures of merit for this disordered structure are actually lower (R1 = 0.0436 and wR2 = 0.1157) than those of the actual structure. Reflections due to the unit cell doubling are however clearly visible in the diffraction pattern and the lower *R* value can be readily explained by the omission of the weaker less accurately determined reflections in the average structure. Satellite reflections caused by the unit cell doubling have an average intensity of 5.8 σ, while all other data average to 18.9 times σ. The structure can thus be seen as a commensurately modulated structure with a q-vector of 0.5 along the *a*-axis direction with the phenyl torsion angles as the only major modulation parameter. Packing of the molecules is unexceptional and partially facilitated by C—H···π interactions (Figure 5) and some slipped π–π stacking, *e.g.* between the ring of C5A through C10A and the double bond of C3B and C4B. The closest contacts, from C10A towards C3B^i^ and C4B^i^, are with 3.374 (2) and 3.295 (2) Å well within the range of substantial π–π interactions (symmetry operator (i): 1 - *x*,-*y*,-*z*). The distance between C10A and the centroid of the double bond is 3.267 Å.

### Experimental

To a solution of 2-phenylimino-3-phenylthiazolidin-4-one (Abdel-Aziz *et al*., 2010) (1, 0.27 g, 1.0 mmol) and benzaldehyde (2, 0.11 g, 1.0 mmol) in ethanol (5.0 ml) was added aqueous potassium carbonate (15%, 3.0 ml) at room temperature. The reaction mixture was stirred at room temperature for 10 h. The progress of the reaction was monitored by TLC using 50% ethyl acetate in hexane as the eluent system. The precipitated solid was filtered, washed with water and dried. The crude product was purified by column chromatography using silica gel and 30% ethyl acetate in hexane as the eluent system to obtain pure product as a white solid. The compound was dissolved in hot methanol and the clear solution was left at room temperature for two days. The crystals formed were filtered off, washed with cold methanol and dried in vacuum. (Yield: 0.21 g, 58.3%; m.p.: 384–386 K; IR (KBr) cm^-1^1680, 1640, 1591, 1370, 1264, 739, 695. ^1^H NMR (CDCl_3_): δ 7.83 (s, 1H), 7.57–7.53 (m, 2H), 7.50–7.34 (m, 10H), 7.19–7.15 (m, 1H), 6.99–6.96 (m, 2H); MS: ESI (*m/z*) 357.3 (*M*+H)^+^.

### Refinement

All hydrogen atoms were added in calculated positions with a C—H bond distance of 0.95 Å and were refined with an isotropic displacement parameters of 1.2 times that of the equivalent isotropic displacement parameter of the adjacent carbon atom.

### Crystal data

**Table:**

C_22_H_16_N_2_OS	*F*(000) = 1488
*M**_r_* = 356.43	*D*_x_ = 1.369 Mg m^−^^3^
Monoclinic, *P*2_1_/*c*	Melting point: 385 K
Hall symbol: -P 2ybc	Mo *K*α radiation, λ = 0.71073 Å
*a* = 10.7814 (9) Å	Cell parameters from 6817 reflections
*b* = 32.779 (3) Å	θ = 2.3–30.7°
*c* = 9.8907 (8) Å	µ = 0.20 mm^−^^1^
β = 98.392 (1)°	*T* = 100 K
*V* = 3458.0 (5) Å^3^	Block, colourless
*Z* = 8	0.55 × 0.41 × 0.33 mm

### Data collection

**Table:**

Bruker SMART APEX CCD diffractometer	10081 independent reflections
Radiation source: fine-focus sealed tube	7732 reflections with *I* > 2σ(*I*)
graphite	*R*_int_ = 0.025
ω scans	θ_max_ = 31.2°, θ_min_ = 2.0°
Absorption correction: multi-scan (*SADABS*; Bruker, 2009)	*h* = −15→15
*T*_min_ = 0.632, *T*_max_ = 0.746	*k* = −47→47
23712 measured reflections	*l* = −14→8

### Refinement

**Table:**

Refinement on *F*^2^	Primary atom site location: structure-invariant direct methods
Least-squares matrix: full	Secondary atom site location: difference Fourier map
*R*[*F*^2^ > 2σ(*F*^2^)] = 0.045	Hydrogen site location: inferred from neighbouring sites
*wR*(*F*^2^) = 0.125	H-atom parameters constrained
*S* = 1.03	*w* = 1/[σ^2^(*F*_o_^2^) + (0.0616*P*)^2^ + 1.0979*P*] where *P* = (*F*_o_^2^ + 2*F*_c_^2^)/3
10081 reflections	(Δ/σ)_max_ < 0.001
469 parameters	Δρ_max_ = 0.46 e Å^−^^3^
0 restraints	Δρ_min_ = −0.23 e Å^−^^3^

### Special details

**Table:**

Geometry. All e.s.d.'s (except the e.s.d. in the dihedral angle between two l.s. planes) are estimated using the full covariance matrix. The cell e.s.d.'s are taken into account individually in the estimation of e.s.d.'s in distances, angles and torsion angles; correlations between e.s.d.'s in cell parameters are only used when they are defined by crystal symmetry. An approximate (isotropic) treatment of cell e.s.d.'s is used for estimating e.s.d.'s involving l.s. planes.
Refinement. Refinement of *F*^2^ against ALL reflections. The weighted *R*-factor *wR* and goodness of fit *S* are based on *F*^2^, conventional *R*-factors *R* are based on *F*, with *F* set to zero for negative *F*^2^. The threshold expression of *F*^2^ > σ(*F*^2^) is used only for calculating *R*-factors(gt) *etc*. and is not relevant to the choice of reflections for refinement. *R*-factors based on *F*^2^ are statistically about twice as large as those based on *F*, and *R*- factors based on ALL data will be even larger.

### Fractional atomic coordinates and isotropic or equivalent isotropic displacement parameters (Å^2^)

**Table:**

	*x*	*y*	*z*	*U*_iso_*/*U*_eq_	
S1A	0.37254 (3)	0.113655 (9)	0.05977 (3)	0.01578 (8)	
O1A	0.20268 (10)	0.06597 (3)	0.33830 (10)	0.0250 (2)	
N1A	0.45402 (11)	0.17270 (3)	0.24759 (11)	0.0165 (2)	
N2A	0.34340 (10)	0.11725 (3)	0.31666 (11)	0.0153 (2)	
C1A	0.39888 (12)	0.13899 (4)	0.22017 (12)	0.0146 (2)	
C2A	0.26620 (13)	0.08498 (4)	0.26862 (13)	0.0172 (2)	
C3A	0.27051 (12)	0.07864 (4)	0.12030 (13)	0.0162 (2)	
C4A	0.19911 (13)	0.04982 (4)	0.05026 (13)	0.0185 (3)	
H4A	0.1531	0.0332	0.1041	0.022*	
C5A	0.18112 (12)	0.03998 (4)	−0.09501 (13)	0.0168 (2)	
C6A	0.23784 (14)	0.06091 (4)	−0.19309 (14)	0.0212 (3)	
H6A	0.2930	0.0829	−0.1656	0.025*	
C7A	0.21457 (14)	0.04992 (5)	−0.32984 (14)	0.0236 (3)	
H7A	0.2545	0.0643	−0.3949	0.028*	
C8A	0.13337 (13)	0.01802 (4)	−0.37254 (14)	0.0215 (3)	
H8A	0.1172	0.0108	−0.4665	0.026*	
C9A	0.07626 (14)	−0.00315 (4)	−0.27756 (14)	0.0235 (3)	
H9A	0.0203	−0.0249	−0.3061	0.028*	
C10A	0.10086 (13)	0.00749 (4)	−0.14036 (14)	0.0211 (3)	
H10A	0.0625	−0.0076	−0.0756	0.025*	
C11A	0.50187 (12)	0.19422 (4)	0.14188 (12)	0.0147 (2)	
C12A	0.59772 (13)	0.17841 (4)	0.07749 (14)	0.0199 (3)	
H12A	0.6301	0.1520	0.1011	0.024*	
C13A	0.64633 (13)	0.20112 (4)	−0.02127 (14)	0.0223 (3)	
H13A	0.7124	0.1903	−0.0642	0.027*	
C14A	0.59849 (14)	0.23952 (4)	−0.05721 (14)	0.0221 (3)	
H14A	0.6312	0.2549	−0.1253	0.026*	
C15A	0.50274 (14)	0.25535 (4)	0.00649 (15)	0.0237 (3)	
H15A	0.4694	0.2815	−0.0188	0.028*	
C16A	0.45523 (13)	0.23312 (4)	0.10705 (14)	0.0204 (3)	
H16A	0.3911	0.2444	0.1520	0.024*	
C17A	0.35528 (12)	0.13144 (4)	0.45589 (12)	0.0152 (2)	
C18A	0.46121 (13)	0.12049 (4)	0.54559 (13)	0.0180 (3)	
H18A	0.5230	0.1032	0.5166	0.022*	
C19A	0.47534 (13)	0.13529 (4)	0.67901 (13)	0.0207 (3)	
H19A	0.5470	0.1279	0.7420	0.025*	
C20A	0.38492 (13)	0.16079 (4)	0.72006 (13)	0.0200 (3)	
H20A	0.3958	0.1713	0.8105	0.024*	
C21A	0.27842 (13)	0.17101 (4)	0.62922 (14)	0.0206 (3)	
H21A	0.2161	0.1880	0.6583	0.025*	
C22A	0.26323 (12)	0.15630 (4)	0.49588 (13)	0.0176 (2)	
H22A	0.1909	0.1632	0.4332	0.021*	
S1B	0.87010 (3)	0.114166 (10)	0.07134 (3)	0.01700 (8)	
O1B	0.68006 (10)	0.07316 (3)	0.34400 (10)	0.0245 (2)	
N1B	0.95837 (11)	0.17253 (3)	0.25832 (11)	0.0172 (2)	
N2B	0.83667 (10)	0.11966 (3)	0.32700 (11)	0.0162 (2)	
C1B	0.89758 (12)	0.13981 (4)	0.23112 (12)	0.0150 (2)	
C2B	0.74959 (13)	0.09027 (4)	0.27595 (13)	0.0174 (2)	
C3B	0.75375 (12)	0.08386 (4)	0.12809 (13)	0.0168 (2)	
C4B	0.67166 (13)	0.05884 (4)	0.05426 (14)	0.0195 (3)	
H4B	0.6119	0.0465	0.1030	0.023*	
C5B	0.66074 (13)	0.04779 (4)	−0.08999 (14)	0.0195 (3)	
C6B	0.75221 (14)	0.05608 (4)	−0.17333 (14)	0.0233 (3)	
H6B	0.8259	0.0706	−0.1370	0.028*	
C7B	0.73604 (16)	0.04329 (5)	−0.30865 (15)	0.0276 (3)	
H7B	0.7981	0.0494	−0.3646	0.033*	
C8B	0.62962 (16)	0.02168 (5)	−0.36239 (15)	0.0299 (3)	
H8B	0.6199	0.0124	−0.4543	0.036*	
C9B	0.53774 (16)	0.01368 (5)	−0.28224 (15)	0.0323 (4)	
H9B	0.4645	−0.0009	−0.3193	0.039*	
C10B	0.55234 (14)	0.02700 (4)	−0.14759 (15)	0.0254 (3)	
H10B	0.4879	0.0219	−0.0938	0.031*	
C11B	1.00800 (12)	0.19307 (4)	0.15183 (13)	0.0162 (2)	
C12B	1.10058 (13)	0.17597 (4)	0.08498 (14)	0.0197 (3)	
H12B	1.1308	0.1493	0.1081	0.024*	
C13B	1.14876 (13)	0.19803 (4)	−0.01569 (14)	0.0225 (3)	
H13B	1.2124	0.1864	−0.0606	0.027*	
C14B	1.10450 (14)	0.23687 (4)	−0.05078 (14)	0.0237 (3)	
H14B	1.1374	0.2518	−0.1199	0.028*	
C15B	1.01203 (15)	0.25394 (4)	0.01530 (15)	0.0251 (3)	
H15B	0.9811	0.2805	−0.0092	0.030*	
C16B	0.96464 (14)	0.23236 (4)	0.11700 (14)	0.0214 (3)	
H16B	0.9024	0.2444	0.1632	0.026*	
C17B	0.84998 (12)	0.13379 (4)	0.46609 (13)	0.0164 (2)	
C18B	0.95529 (13)	0.12222 (4)	0.55533 (14)	0.0203 (3)	
H18B	1.0158	0.1045	0.5260	0.024*	
C19B	0.97085 (13)	0.13698 (4)	0.68874 (14)	0.0229 (3)	
H19B	1.0423	0.1293	0.7514	0.027*	
C20B	0.88194 (13)	0.16298 (4)	0.73020 (14)	0.0216 (3)	
H20B	0.8935	0.1733	0.8209	0.026*	
C21B	0.77625 (13)	0.17394 (4)	0.63987 (14)	0.0215 (3)	
H21B	0.7153	0.1915	0.6691	0.026*	
C22B	0.75968 (13)	0.15919 (4)	0.50639 (14)	0.0192 (3)	
H22B	0.6875	0.1664	0.4440	0.023*	

### Atomic displacement parameters (Å^2^)

**Table:**

	*U*^11^	*U*^22^	*U*^33^	*U*^12^	*U*^13^	*U*^23^
S1A	0.01872 (16)	0.01662 (15)	0.01241 (15)	−0.00329 (11)	0.00371 (11)	−0.00114 (10)
O1A	0.0353 (6)	0.0255 (5)	0.0152 (5)	−0.0138 (4)	0.0071 (4)	−0.0009 (4)
N1A	0.0200 (6)	0.0162 (5)	0.0136 (5)	−0.0024 (4)	0.0030 (4)	0.0006 (4)
N2A	0.0190 (5)	0.0150 (5)	0.0122 (5)	−0.0037 (4)	0.0030 (4)	0.0003 (4)
C1A	0.0159 (6)	0.0158 (5)	0.0121 (6)	0.0010 (4)	0.0019 (4)	0.0009 (4)
C2A	0.0212 (6)	0.0158 (5)	0.0145 (6)	−0.0023 (5)	0.0023 (5)	0.0004 (4)
C3A	0.0194 (6)	0.0152 (5)	0.0142 (6)	−0.0018 (5)	0.0032 (5)	0.0009 (4)
C4A	0.0229 (7)	0.0184 (6)	0.0146 (6)	−0.0044 (5)	0.0034 (5)	0.0004 (5)
C5A	0.0182 (6)	0.0174 (6)	0.0146 (6)	−0.0008 (5)	0.0016 (5)	−0.0010 (4)
C6A	0.0262 (7)	0.0214 (6)	0.0159 (6)	−0.0055 (5)	0.0025 (5)	−0.0007 (5)
C7A	0.0270 (7)	0.0292 (7)	0.0149 (6)	−0.0051 (6)	0.0038 (5)	0.0006 (5)
C8A	0.0227 (7)	0.0274 (7)	0.0134 (6)	0.0015 (5)	−0.0010 (5)	−0.0030 (5)
C9A	0.0247 (7)	0.0238 (7)	0.0211 (7)	−0.0040 (5)	0.0003 (5)	−0.0054 (5)
C10A	0.0233 (7)	0.0214 (6)	0.0187 (7)	−0.0043 (5)	0.0038 (5)	−0.0023 (5)
C11A	0.0164 (6)	0.0150 (5)	0.0123 (6)	−0.0035 (4)	0.0009 (4)	−0.0006 (4)
C12A	0.0196 (6)	0.0200 (6)	0.0203 (7)	0.0013 (5)	0.0036 (5)	0.0028 (5)
C13A	0.0201 (7)	0.0265 (7)	0.0212 (7)	−0.0006 (5)	0.0058 (5)	0.0010 (5)
C14A	0.0246 (7)	0.0244 (7)	0.0171 (7)	−0.0063 (5)	0.0025 (5)	0.0045 (5)
C15A	0.0279 (7)	0.0180 (6)	0.0251 (7)	−0.0002 (5)	0.0034 (6)	0.0055 (5)
C16A	0.0229 (7)	0.0178 (6)	0.0211 (7)	0.0001 (5)	0.0057 (5)	0.0004 (5)
C17A	0.0194 (6)	0.0155 (5)	0.0107 (6)	−0.0041 (5)	0.0023 (4)	−0.0002 (4)
C18A	0.0179 (6)	0.0192 (6)	0.0168 (6)	−0.0003 (5)	0.0024 (5)	0.0019 (5)
C19A	0.0199 (7)	0.0267 (7)	0.0146 (6)	−0.0033 (5)	−0.0007 (5)	0.0019 (5)
C20A	0.0253 (7)	0.0227 (6)	0.0123 (6)	−0.0071 (5)	0.0034 (5)	−0.0006 (5)
C21A	0.0220 (7)	0.0229 (6)	0.0180 (7)	−0.0021 (5)	0.0070 (5)	−0.0024 (5)
C22A	0.0173 (6)	0.0192 (6)	0.0162 (6)	−0.0015 (5)	0.0018 (5)	0.0007 (5)
S1B	0.01907 (17)	0.01822 (15)	0.01406 (16)	−0.00326 (11)	0.00352 (12)	−0.00196 (11)
O1B	0.0306 (6)	0.0250 (5)	0.0192 (5)	−0.0105 (4)	0.0082 (4)	−0.0019 (4)
N1B	0.0190 (5)	0.0175 (5)	0.0152 (5)	−0.0015 (4)	0.0025 (4)	0.0002 (4)
N2B	0.0185 (5)	0.0175 (5)	0.0130 (5)	−0.0028 (4)	0.0031 (4)	−0.0006 (4)
C1B	0.0151 (6)	0.0171 (5)	0.0126 (6)	0.0011 (4)	0.0016 (4)	0.0000 (4)
C2B	0.0198 (6)	0.0153 (5)	0.0169 (6)	−0.0017 (5)	0.0027 (5)	−0.0011 (4)
C3B	0.0191 (6)	0.0161 (5)	0.0156 (6)	−0.0014 (5)	0.0039 (5)	−0.0009 (4)
C4B	0.0214 (7)	0.0178 (6)	0.0193 (7)	−0.0028 (5)	0.0035 (5)	−0.0006 (5)
C5B	0.0236 (7)	0.0170 (6)	0.0172 (6)	−0.0012 (5)	0.0003 (5)	−0.0004 (5)
C6B	0.0258 (7)	0.0234 (6)	0.0208 (7)	−0.0048 (5)	0.0035 (5)	−0.0043 (5)
C7B	0.0349 (9)	0.0298 (7)	0.0188 (7)	−0.0031 (6)	0.0065 (6)	−0.0029 (6)
C8B	0.0370 (9)	0.0350 (8)	0.0156 (7)	−0.0032 (7)	−0.0028 (6)	−0.0031 (6)
C9B	0.0336 (9)	0.0403 (9)	0.0202 (7)	−0.0108 (7)	−0.0054 (6)	−0.0034 (6)
C10B	0.0266 (8)	0.0277 (7)	0.0207 (7)	−0.0041 (6)	−0.0009 (6)	0.0011 (5)
C11B	0.0169 (6)	0.0171 (5)	0.0139 (6)	−0.0042 (5)	0.0004 (5)	−0.0004 (4)
C12B	0.0181 (6)	0.0201 (6)	0.0208 (7)	0.0008 (5)	0.0021 (5)	0.0028 (5)
C13B	0.0184 (7)	0.0284 (7)	0.0209 (7)	−0.0006 (5)	0.0040 (5)	0.0012 (5)
C14B	0.0243 (7)	0.0271 (7)	0.0190 (7)	−0.0066 (6)	0.0012 (5)	0.0054 (5)
C15B	0.0302 (8)	0.0189 (6)	0.0257 (7)	−0.0014 (5)	0.0020 (6)	0.0046 (5)
C16B	0.0233 (7)	0.0189 (6)	0.0222 (7)	0.0002 (5)	0.0043 (5)	0.0001 (5)
C17B	0.0196 (6)	0.0171 (6)	0.0126 (6)	−0.0041 (5)	0.0027 (5)	−0.0001 (4)
C18B	0.0198 (7)	0.0226 (6)	0.0182 (7)	0.0003 (5)	0.0019 (5)	0.0000 (5)
C19B	0.0213 (7)	0.0291 (7)	0.0171 (7)	−0.0021 (5)	−0.0012 (5)	0.0021 (5)
C20B	0.0235 (7)	0.0270 (7)	0.0142 (6)	−0.0068 (5)	0.0028 (5)	−0.0019 (5)
C21B	0.0212 (7)	0.0260 (7)	0.0180 (7)	−0.0008 (5)	0.0056 (5)	−0.0022 (5)
C22B	0.0181 (6)	0.0221 (6)	0.0171 (6)	−0.0008 (5)	0.0023 (5)	−0.0001 (5)

### Geometric parameters (Å, °)

**Table:**

S1A—C3A	1.7544 (13)	S1B—C3B	1.7553 (13)
S1A—C1A	1.7764 (13)	S1B—C1B	1.7762 (13)
O1A—C2A	1.2124 (15)	O1B—C2B	1.2151 (16)
N1A—C1A	1.2653 (16)	N1B—C1B	1.2654 (16)
N1A—C11A	1.4192 (16)	N1B—C11B	1.4189 (16)
N2A—C2A	1.3863 (16)	N2B—C2B	1.3879 (16)
N2A—C1A	1.3940 (16)	N2B—C1B	1.3960 (16)
N2A—C17A	1.4414 (15)	N2B—C17B	1.4387 (16)
C2A—C3A	1.4891 (17)	C2B—C3B	1.4846 (18)
C3A—C4A	1.3452 (17)	C3B—C4B	1.3418 (18)
C4A—C5A	1.4577 (18)	C4B—C5B	1.4600 (18)
C4A—H4A	0.9500	C4B—H4B	0.9500
C5A—C6A	1.3997 (18)	C5B—C10B	1.4003 (19)
C5A—C10A	1.4039 (18)	C5B—C6B	1.401 (2)
C6A—C7A	1.3867 (19)	C6B—C7B	1.389 (2)
C6A—H6A	0.9500	C6B—H6B	0.9500
C7A—C8A	1.3893 (19)	C7B—C8B	1.387 (2)
C7A—H7A	0.9500	C7B—H7B	0.9500
C8A—C9A	1.383 (2)	C8B—C9B	1.381 (2)
C8A—H8A	0.9500	C8B—H8B	0.9500
C9A—C10A	1.3887 (19)	C9B—C10B	1.388 (2)
C9A—H9A	0.9500	C9B—H9B	0.9500
C10A—H10A	0.9500	C10B—H10B	0.9500
C11A—C12A	1.3909 (18)	C11B—C12B	1.3930 (19)
C11A—C16A	1.3952 (18)	C11B—C16B	1.3960 (18)
C12A—C13A	1.3902 (19)	C12B—C13B	1.3912 (19)
C12A—H12A	0.9500	C12B—H12B	0.9500
C13A—C14A	1.387 (2)	C13B—C14B	1.386 (2)
C13A—H13A	0.9500	C13B—H13B	0.9500
C14A—C15A	1.386 (2)	C14B—C15B	1.387 (2)
C14A—H14A	0.9500	C14B—H14B	0.9500
C15A—C16A	1.3898 (19)	C15B—C16B	1.3868 (19)
C15A—H15A	0.9500	C15B—H15B	0.9500
C16A—H16A	0.9500	C16B—H16B	0.9500
C17A—C22A	1.3855 (18)	C17B—C22B	1.3831 (19)
C17A—C18A	1.3873 (18)	C17B—C18B	1.3854 (18)
C18A—C19A	1.3933 (18)	C18B—C19B	1.3925 (19)
C18A—H18A	0.9500	C18B—H18B	0.9500
C19A—C20A	1.389 (2)	C19B—C20B	1.389 (2)
C19A—H19A	0.9500	C19B—H19B	0.9500
C20A—C21A	1.3917 (19)	C20B—C21B	1.389 (2)
C20A—H20A	0.9500	C20B—H20B	0.9500
C21A—C22A	1.3914 (18)	C21B—C22B	1.3928 (18)
C21A—H21A	0.9500	C21B—H21B	0.9500
C22A—H22A	0.9500	C22B—H22B	0.9500
			
C3A—S1A—C1A	91.58 (6)	C3B—S1B—C1B	91.01 (6)
C1A—N1A—C11A	119.08 (11)	C1B—N1B—C11B	118.95 (11)
C2A—N2A—C1A	116.85 (10)	C2B—N2B—C1B	116.21 (10)
C2A—N2A—C17A	122.60 (10)	C2B—N2B—C17B	122.62 (11)
C1A—N2A—C17A	120.13 (10)	C1B—N2B—C17B	120.41 (10)
N1A—C1A—N2A	122.34 (11)	N1B—C1B—N2B	122.48 (11)
N1A—C1A—S1A	127.49 (10)	N1B—C1B—S1B	127.18 (10)
N2A—C1A—S1A	110.10 (9)	N2B—C1B—S1B	110.28 (9)
O1A—C2A—N2A	123.92 (12)	O1B—C2B—N2B	123.92 (12)
O1A—C2A—C3A	126.13 (12)	O1B—C2B—C3B	126.16 (12)
N2A—C2A—C3A	109.91 (11)	N2B—C2B—C3B	109.91 (11)
C4A—C3A—C2A	120.50 (12)	C4B—C3B—C2B	120.68 (12)
C4A—C3A—S1A	128.61 (10)	C4B—C3B—S1B	128.12 (10)
C2A—C3A—S1A	110.86 (9)	C2B—C3B—S1B	111.14 (9)
C3A—C4A—C5A	130.30 (12)	C3B—C4B—C5B	129.51 (13)
C3A—C4A—H4A	114.8	C3B—C4B—H4B	115.2
C5A—C4A—H4A	114.8	C5B—C4B—H4B	115.2
C6A—C5A—C10A	117.50 (12)	C10B—C5B—C6B	118.19 (13)
C6A—C5A—C4A	124.47 (12)	C10B—C5B—C4B	117.50 (13)
C10A—C5A—C4A	118.03 (12)	C6B—C5B—C4B	124.30 (13)
C7A—C6A—C5A	120.90 (13)	C7B—C6B—C5B	120.54 (14)
C7A—C6A—H6A	119.6	C7B—C6B—H6B	119.7
C5A—C6A—H6A	119.6	C5B—C6B—H6B	119.7
C6A—C7A—C8A	120.55 (13)	C8B—C7B—C6B	120.27 (14)
C6A—C7A—H7A	119.7	C8B—C7B—H7B	119.9
C8A—C7A—H7A	119.7	C6B—C7B—H7B	119.9
C9A—C8A—C7A	119.64 (12)	C9B—C8B—C7B	119.97 (14)
C9A—C8A—H8A	120.2	C9B—C8B—H8B	120.0
C7A—C8A—H8A	120.2	C7B—C8B—H8B	120.0
C8A—C9A—C10A	119.83 (13)	C8B—C9B—C10B	120.04 (14)
C8A—C9A—H9A	120.1	C8B—C9B—H9B	120.0
C10A—C9A—H9A	120.1	C10B—C9B—H9B	120.0
C9A—C10A—C5A	121.57 (13)	C9B—C10B—C5B	120.95 (14)
C9A—C10A—H10A	119.2	C9B—C10B—H10B	119.5
C5A—C10A—H10A	119.2	C5B—C10B—H10B	119.5
C12A—C11A—C16A	119.51 (12)	C12B—C11B—C16B	119.44 (12)
C12A—C11A—N1A	121.76 (11)	C12B—C11B—N1B	122.37 (12)
C16A—C11A—N1A	118.65 (11)	C16B—C11B—N1B	118.16 (12)
C13A—C12A—C11A	120.24 (12)	C13B—C12B—C11B	119.92 (13)
C13A—C12A—H12A	119.9	C13B—C12B—H12B	120.0
C11A—C12A—H12A	119.9	C11B—C12B—H12B	120.0
C14A—C13A—C12A	120.13 (13)	C14B—C13B—C12B	120.39 (13)
C14A—C13A—H13A	119.9	C14B—C13B—H13B	119.8
C12A—C13A—H13A	119.9	C12B—C13B—H13B	119.8
C15A—C14A—C13A	119.81 (13)	C13B—C14B—C15B	119.84 (13)
C15A—C14A—H14A	120.1	C13B—C14B—H14B	120.1
C13A—C14A—H14A	120.1	C15B—C14B—H14B	120.1
C14A—C15A—C16A	120.37 (13)	C16B—C15B—C14B	120.13 (13)
C14A—C15A—H15A	119.8	C16B—C15B—H15B	119.9
C16A—C15A—H15A	119.8	C14B—C15B—H15B	119.9
C15A—C16A—C11A	119.92 (13)	C15B—C16B—C11B	120.27 (13)
C15A—C16A—H16A	120.0	C15B—C16B—H16B	119.9
C11A—C16A—H16A	120.0	C11B—C16B—H16B	119.9
C22A—C17A—C18A	121.78 (12)	C22B—C17B—C18B	121.81 (12)
C22A—C17A—N2A	119.68 (11)	C22B—C17B—N2B	119.55 (12)
C18A—C17A—N2A	118.52 (11)	C18B—C17B—N2B	118.63 (12)
C17A—C18A—C19A	118.80 (12)	C17B—C18B—C19B	118.86 (13)
C17A—C18A—H18A	120.6	C17B—C18B—H18B	120.6
C19A—C18A—H18A	120.6	C19B—C18B—H18B	120.6
C20A—C19A—C18A	120.13 (13)	C20B—C19B—C18B	120.02 (13)
C20A—C19A—H19A	119.9	C20B—C19B—H19B	120.0
C18A—C19A—H19A	119.9	C18B—C19B—H19B	120.0
C19A—C20A—C21A	120.29 (12)	C19B—C20B—C21B	120.38 (13)
C19A—C20A—H20A	119.9	C19B—C20B—H20B	119.8
C21A—C20A—H20A	119.9	C21B—C20B—H20B	119.8
C22A—C21A—C20A	120.02 (13)	C20B—C21B—C22B	119.97 (13)
C22A—C21A—H21A	120.0	C20B—C21B—H21B	120.0
C20A—C21A—H21A	120.0	C22B—C21B—H21B	120.0
C17A—C22A—C21A	118.97 (12)	C17B—C22B—C21B	118.94 (13)
C17A—C22A—H22A	120.5	C17B—C22B—H22B	120.5
C21A—C22A—H22A	120.5	C21B—C22B—H22B	120.5
			
C11A—N1A—C1A—N2A	−176.35 (11)	C11B—N1B—C1B—N2B	−174.35 (11)
C11A—N1A—C1A—S1A	0.28 (18)	C11B—N1B—C1B—S1B	2.64 (18)
C2A—N2A—C1A—N1A	168.24 (12)	C2B—N2B—C1B—N1B	164.86 (12)
C17A—N2A—C1A—N1A	−4.49 (19)	C17B—N2B—C1B—N1B	−5.38 (19)
C2A—N2A—C1A—S1A	−8.91 (14)	C2B—N2B—C1B—S1B	−12.58 (14)
C17A—N2A—C1A—S1A	178.36 (9)	C17B—N2B—C1B—S1B	177.18 (9)
C3A—S1A—C1A—N1A	−169.45 (13)	C3B—S1B—C1B—N1B	−166.12 (13)
C3A—S1A—C1A—N2A	7.52 (10)	C3B—S1B—C1B—N2B	11.17 (10)
C1A—N2A—C2A—O1A	−172.66 (13)	C1B—N2B—C2B—O1B	−172.17 (13)
C17A—N2A—C2A—O1A	−0.1 (2)	C17B—N2B—C2B—O1B	−2.2 (2)
C1A—N2A—C2A—C3A	5.38 (16)	C1B—N2B—C2B—C3B	6.89 (16)
C17A—N2A—C2A—C3A	177.92 (11)	C17B—N2B—C2B—C3B	176.89 (11)
O1A—C2A—C3A—C4A	0.5 (2)	O1B—C2B—C3B—C4B	3.7 (2)
N2A—C2A—C3A—C4A	−177.54 (12)	N2B—C2B—C3B—C4B	−175.32 (12)
O1A—C2A—C3A—S1A	178.68 (12)	O1B—C2B—C3B—S1B	−178.94 (12)
N2A—C2A—C3A—S1A	0.69 (14)	N2B—C2B—C3B—S1B	2.02 (14)
C1A—S1A—C3A—C4A	173.38 (13)	C1B—S1B—C3B—C4B	169.61 (13)
C1A—S1A—C3A—C2A	−4.67 (10)	C1B—S1B—C3B—C2B	−7.49 (10)
C2A—C3A—C4A—C5A	175.30 (13)	C2B—C3B—C4B—C5B	−178.83 (13)
S1A—C3A—C4A—C5A	−2.6 (2)	S1B—C3B—C4B—C5B	4.3 (2)
C3A—C4A—C5A—C6A	−1.2 (2)	C3B—C4B—C5B—C10B	−168.33 (14)
C3A—C4A—C5A—C10A	179.25 (14)	C3B—C4B—C5B—C6B	12.7 (2)
C10A—C5A—C6A—C7A	0.4 (2)	C10B—C5B—C6B—C7B	−1.1 (2)
C4A—C5A—C6A—C7A	−179.19 (13)	C4B—C5B—C6B—C7B	177.88 (13)
C5A—C6A—C7A—C8A	0.6 (2)	C5B—C6B—C7B—C8B	−0.8 (2)
C6A—C7A—C8A—C9A	−0.6 (2)	C6B—C7B—C8B—C9B	1.6 (2)
C7A—C8A—C9A—C10A	−0.3 (2)	C7B—C8B—C9B—C10B	−0.5 (3)
C8A—C9A—C10A—C5A	1.2 (2)	C8B—C9B—C10B—C5B	−1.5 (2)
C6A—C5A—C10A—C9A	−1.3 (2)	C6B—C5B—C10B—C9B	2.2 (2)
C4A—C5A—C10A—C9A	178.32 (13)	C4B—C5B—C10B—C9B	−176.82 (14)
C1A—N1A—C11A—C12A	−64.30 (17)	C1B—N1B—C11B—C12B	−64.65 (17)
C1A—N1A—C11A—C16A	118.93 (14)	C1B—N1B—C11B—C16B	117.54 (14)
C16A—C11A—C12A—C13A	−0.3 (2)	C16B—C11B—C12B—C13B	−0.1 (2)
N1A—C11A—C12A—C13A	−177.02 (12)	N1B—C11B—C12B—C13B	−177.89 (12)
C11A—C12A—C13A—C14A	−0.7 (2)	C11B—C12B—C13B—C14B	−0.5 (2)
C12A—C13A—C14A—C15A	0.6 (2)	C12B—C13B—C14B—C15B	0.3 (2)
C13A—C14A—C15A—C16A	0.6 (2)	C13B—C14B—C15B—C16B	0.5 (2)
C14A—C15A—C16A—C11A	−1.6 (2)	C14B—C15B—C16B—C11B	−1.1 (2)
C12A—C11A—C16A—C15A	1.4 (2)	C12B—C11B—C16B—C15B	0.9 (2)
N1A—C11A—C16A—C15A	178.27 (12)	N1B—C11B—C16B—C15B	178.82 (12)
C2A—N2A—C17A—C22A	−79.01 (16)	C2B—N2B—C17B—C22B	−73.31 (16)
C1A—N2A—C17A—C22A	93.29 (15)	C1B—N2B—C17B—C22B	96.28 (15)
C2A—N2A—C17A—C18A	102.61 (15)	C2B—N2B—C17B—C18B	108.23 (15)
C1A—N2A—C17A—C18A	−85.09 (15)	C1B—N2B—C17B—C18B	−82.17 (16)
C22A—C17A—C18A—C19A	−0.57 (19)	C22B—C17B—C18B—C19B	−0.7 (2)
N2A—C17A—C18A—C19A	177.78 (11)	N2B—C17B—C18B—C19B	177.73 (12)
C17A—C18A—C19A—C20A	−0.5 (2)	C17B—C18B—C19B—C20B	−0.3 (2)
C18A—C19A—C20A—C21A	1.4 (2)	C18B—C19B—C20B—C21B	1.0 (2)
C19A—C20A—C21A—C22A	−1.2 (2)	C19B—C20B—C21B—C22B	−0.7 (2)
C18A—C17A—C22A—C21A	0.78 (19)	C18B—C17B—C22B—C21B	0.9 (2)
N2A—C17A—C22A—C21A	−177.56 (11)	N2B—C17B—C22B—C21B	−177.47 (12)
C20A—C21A—C22A—C17A	0.1 (2)	C20B—C21B—C22B—C17B	−0.2 (2)

### Hydrogen-bond geometry (Å, °)

**Table:**

*D*—H···*A*	*D*—H	H···*A*	*D*···*A*	*D*—H···*A*
C12A—H12A···S1B	0.95	2.92	3.6214 (15)	131
C12A—H12A···C2B	0.95	2.85	3.7340 (19)	156
C12A—H12A···C3B	0.95	2.59	3.5270 (19)	167
C12B—H12B···S1A^i^	0.95	2.96	3.6118 (15)	127
C12B—H12B···C3A^i^	0.95	2.76	3.6700 (19)	162

Symmetry codes: (i) *x*+1, *y*, *z*.
